# Prevalence and Treatment Outcome of Nasolacrimal Duct Obstruction in Saudi Children with Down Syndrome

**DOI:** 10.7759/cureus.6672

**Published:** 2020-01-15

**Authors:** Lujain Abdu, Noor Bawahab, Raghad W Mohammed Hussain, Hesham Qary, Asalh Saeedi, Nizar Alhibshi

**Affiliations:** 1 Medicine, King Abdulaziz University, Jeddah, SAU; 2 Pediatric, King Abdulaziz University Hospital, Jeddah, SAU; 3 Pediatrics, King Abdulaziz University Hospital, Jeddah, SAU; 4 Ophthalmology, King Abdulaziz University Hospital, Jeddah, SAU

**Keywords:** down syndrome, nasolacrimal duct, ophthalmology, genetic, pediatric

## Abstract

Introduction

Congenital nasolacrimal duct obstruction (CNLDO) is one of the most common congenital abnormalities encountered by pediatric ophthalmologists, occurring in 20-30% of all neonates (range: 6-84%). The majority of the cases (up to 90%) resolve within the first year of birth. Many syndromes, such as Down syndrome, are associated with congenital lacrimal anomalies. The prevalence of nasolacrimal anomalies in Down syndrome has been reported to be 22%.

Methods

This was a retrospective study of all children diagnosed with Down syndrome at King Abdulaziz University Hospital (KAUH), Jeddah, Saudi Arabia between 2010 and 2015.

Result

The total sample size was 175 patients; 15 patients were diagnosed with CNLDO with a prevalence of 8.57%. The prevalence among the gender was 53.3% male and 46.7% female, with a median age of eight years. Regarding ocular disorders, 20.0% cases were diagnosed with refractive error, 13.3% with nystagmus, and 13.3% with blepharitis. Myopia, strabismus, conjunctivitis, and cataract were observed in four different patients, and the remaining four cases reported no other ocular disorders. Tearing, alone or associated with other symptoms, was the main presentation of CNLDO (86.7%). Bilateral CNLDO was the most commonly observed abnormality, alone or associated with others. The median age at diagnosis was one year. Of the cases, 53.3% were treated medically, 26.7% by surgical correction, and 13.3% required both.

Conclusion

Bilateral CNLDO is the most observed disease pattern among children with Down syndrome. In our study, tearing was the most common clinical presentation and most cases were treated medically. Down syndrome patients should be carefully examined for nasolacrimal duct obstructions and treated medically.

## Introduction

Congenital nasolacrimal duct obstruction (CNLDO) is one of the most common congenital abnormalities encountered by pediatric ophthalmologists, occurring in 20-30% of all neonates (range: 6-84%) [1-4]. It results from an obstruction at the valve of the Hasner membrane, and despite its high global rates of incidence, it clinically becomes symptomatic in only 2-6% of all affected infants [3,5]. Furthermore, the majority of the cases (up to 90%) resolve within the first year of birth [6-7].

Several risk factors are associated with CNLDO, such as maternal infections during pregnancy, medication use, radiation exposure, some occupational hazards, and genetic predisposition [8].

Several studies have confirmed that amblyopia, also known as lazy eye, is one of the main complications caused by CNLDO, developing in approximately 22% of the children diagnosed with CNLDO [6,9-11]. Presenting features of CNLDO include constant epiphora and intermittent discharge involving one or both eyes [3,12]. In the majority of cases, it is considered a benign disease as far as visual development is concerned [3]. Normal development of the visual system early in life requires the presence of a sharply focused retinal image. The effect of persistent tearing on visual development in children is debatable and inconclusive. Hypothetically, continuous watering due to CNLDO can lead to vision cloudiness and amblyopia during visual development [3].

Many syndromes are known to be associated with congenital lacrimal anomalies. The most common are Down syndrome and ectrodactyly ectodermal dysplasia-cleft (EEC) syndrome [13-16]. The prevalence of nasolacrimal anomalies in Down syndrome has been reported to be 22% [13-14]. The prevalence of CNLDO in Saudi Arabia is estimated to be 2.6% [17]. The literature concerning Saudi Arabia does not yet report on the prevalence of CNLDO in Down syndrome patients or the outcomes of different treatment options. Our study aims to describe the prevalence and clinical presentation of CNLDO among children diagnosed with Down syndrome and the treatment outcomes in a tertiary care centre in Saudi Arabia.

## Materials and methods

This was a retrospective review including all children diagnosed with Down syndrome at King Abdulaziz University Hospital (KAUH), Jeddah, Saudi Arabia between 2010 and 2015. Ethical approval was obtained from the technical and ethical committee at KAUH, as well as other administrative approvals from KAUH administration.

The records of pediatric patients diagnosed with Down syndrome per national guidelines within the above years were reviewed. We used a standardized data collection sheet designed from literature findings to collect the following patient information: (A) Demographic data (age, gender, and nationality), (B) Clinical profile (past medical history of other ocular disorders and chronic systemic conditions), (C) CNLDO presentation (age at presentation, symptoms and signs, disease pattern), and (D) CNLDO management and prognosis (prescribed medical treatments, type of surgical correction, and final treatment outcome).

Patients’ personal information was kept confidential, and obtained data were coded and sorted for analysis. Descriptive statistical analysis was conducted using the Statistical Package for Social Sciences (SPSS) for Windows Version 22 (IBM Corp., Armonk, NY). Categorical data were represented in counts and frequencies, whereas continuous data were described in ranges and medians.

## Results

The total sample size was 175 patients; 15 patients were diagnosed with CNLDO with prevalence of 8.57%. Eight (53.3%) males and seven (46.7%) females, with a median age of eight years, were included in this review. Ten (66.7%) of these patients were Saudi children (Table 1).

**Table 1:** Demographic data

Variable	N	%
Gender		
Male	7	46.7
Female	8	53.3
Nationality		
Saudi	10	66.7
Non-Saudi	5	33.3
Variable	Median	Quartile (25-75)
Age	8	(6-9)

Regarding ocular disorders, three (20.0%) cases were diagnosed with refractive error, two had nystagmus (13.3%), and two cases had blepharitis (13.3%). Myopia, strabismus, conjunctivitis, and cataract were observed in four different patients, and the remaining four cases reported no other ocular disorders. In terms of systemic diseases, congenital heart diseases were reported in four (18.2%) cases, thyroid disorders were also reported in four (18.2%) cases, atrial septal defects were reported in three (13.6%) cases, and kidney diseases and neurological diseases were each reported in two (9.1%) cases, whereas the remaining four (18.2%) cases had no known systemic diseases (Table 2). Tearing, alone or associated with other symptoms, was the main presentation of CNLDO in 13 (86.7%) cases. Bilateral CNLDO was the most commonly observed abnormality, alone or associated with others (Table 2). The median age at diagnosis was one year.

**Table 2:** Clinical characteristics

Variable	N	%
Ocular diseases		
Refractive error	3	20.0
Nystagmus	2	13.3
Blepharitis	2	13.3
Myopia	1	6.7
Strabismus	1	6.7
Conjunctivitis	1	6.7
Cataract	1	6.7
No ocular disease	4	26.7
Systematic diseases		
None	4	18.2
Congenital heart disease	4	18.2
Atrial septal defect	3	13.6
Other heart diseases	3	13.6
Thyroid disorder	4	18.2
Kidney disease	2	9.1
Neurological diseases	2	9.1
Symptoms		
Tearing; discharge in the eye	7	46.7
Discharge in the eye	2	13.3
Tearing	5	33.3
Tearing; discharge in the eye; Sleep with eyes open	1	6.7
Abnormalities		
Bilateral	9	60.0
Bilateral; recurrent; tight nasolacrimal duct	2	13.3
Upward slanting of the palpebral fissure - epicanthal folds	1	6.7
Unilateral; Right eye	1	6.7
Bilateral; recurrent	2	13.3

Various treatment modalities were used for these patients. Medical treatment was used in eight (53.3%) cases, followed by surgical correction in four (26.7%) cases, and two (13.3%) cases required both. The main medical treatment used in eight (53.3%) cases was “Massage”, while the main surgical method applied in three (20.0%) cases was “Probing”. Complete resolution was reported in nine (60.0%) cases, and three cases reported a partial resolution.

## Discussion

Down syndrome, a chromosomal anomaly caused by trisomy 21, is one of the most common congenital anomalies. Several studies have reported its association with a number of ophthalmic features where the lacrimal drainage system is often influenced [13,17-18]. The incidence rate of nasolacrimal duct obstruction ranges between 5% and 30% [13,19].

The nasolacrimal duct is formed by canalization of the caudal extremity of an epithelial cord derived from the ectoderm in the naso-optic fissure, which is often not completed at birth [1]. If there is a failure in the canalization of the nasolacrimal duct, particularly on the membrane of Hanser, CNLDO will occur [20]. There are three main clinical manifestations of CNLDO: persistent epiphora, increased tear lake, and recurrent mucopurulent discharge [20]. In the current study, tearing with or without discharge in the eye was the main symptom in all the cases.

Patients with Down syndrome that have CNLDO have anatomical abnormalities either as focal stenosis or diffuse stenosis, other than obstructions at its distal end [12]. The higher rate of CNLDO in Down syndrome could be explained in part to the unique facial morphology, and abnormal persistence of a membrane or bony obstruction along the distal portion of the nasolacrimal duct (NLD) [21].

There are two major treatment approaches for CNLDO, medically and surgically. In the current study, more than half of the patients were treated medically, mainly by massage. About a fourth of the patients were treated surgically, mainly by probing.

In a Brazilian study, most of the ophthalmologists (97.2%) stated they used massage as the primary treatment approach until the age of one year [22]. Similar results were reported in a UK study, where 84.0% of the ophthalmologists used massage as the primary approach until the age of one year [23]. The “wait-and-see” model associated with conservative therapies was confirmed in many studies to be a better treatment option in infants less than one year old [22-25].

As a patient ages, the rate of spontaneous resolution of CNLDO reduces, and surgical intervention, such as probing, must be performed. Probing can be performed under general anesthesia or sedation. In the Brazilian and UK studies, the procedure was performed under anesthesia in patients between the ages of 12-15 months [22-23]. Probing is considered the first surgical option because of its ease of implementation [22].

The optimal timing for probing remains debatable. Several studies have recommended early probing, and the reason is that prolonged inflammation raises fibrosis in the obstructed sites. Moreover, Arora et al. concluded that probing with irrigation is considered primary management for congenital NLDO in children less than three years old. Based on their data and the results of many other studies, the failing of probing with irrigation as primary treatment is likely with children beyond age three along with other clinical factors [26-27]. On the other hand, other studies have reported probing to be an applicable surgical option for children between two and three years of age who present with primary CNLDO [28-30].

Our study limitations included retrospective design and varying follow-up duration.

## Conclusions

The prevalence of CNLDO in Down syndrome patients was 8.57%. Bilateral CNLDO is the most observed disease pattern among children with Down syndrome, with tearing as the most common clinical presentation. Patients are mostly treated medically. Therefore, we recommend that Down syndrome patients should be carefully examined for nasolacrimal duct obstructions.
